# Promoting primary palliative care in Western Kenya using Project ECHO^®^

**DOI:** 10.4102/phcfm.v17i1.5138

**Published:** 2025-11-24

**Authors:** Hussein Elias, Sarah Nyariki, Caitrin M. Kelly, Terry Vik, Kenneth Cornetta

**Affiliations:** 1 Department of Family Medicine, Medical Education and Community Health, School of Medicine, Moi University, Eldoret, Kenya; 2 Academic Model Providing Access to Healthcare, Eldoret, Kenya; 3 Department of Medicine, School of Medicine, Indiana University, Indianapolis, United States of America; 4 Division of Pediatric Hemato-oncology, School of Medicine, Indiana University, Indianapolis, United States of America; 5 Department of Medical and Molecular Genetics, School of Medicine, Indiana University, Indianapolis, United States of America

**Keywords:** primary care, palliative care, education, rural, virtual, hub and spoke

## Abstract

Currently less than 2% of Kenyans with severe symptoms receive palliative care (PC). Moreover, PC services are concentrated in urban settings and most rural healthcare providers have limited PC expertise. Project ECHO^®^ Palliative Care for Western Kenya was developed as part of a hub-and-spoke model for improving primary PC in rural Kenya. The programme is based at Moi University and Moi Teaching and Referral Hospital, a public, tertiary care facility with a catchment of 25 million Kenyans, the majority of whom live in rural settings. Self-reported assessments by primary care providers found the Project ECHO® Palliative Care for Western Kenya programme improved PC knowledge and clinical skills, increased professional confidence and decreased professional isolation. The training sessions led to an increase in collaborative care management between primary care providers and PC specialists outside of the educational sessions. While a positive finding, it does present challenges to an already small cadre of PC specialists in Western Kenya. A monthly education programme is a useful tool for expanding primary PC services, but optimal clinical care will require increasing the number of speciality PC providers. Effective PC will be most effective when primary and speciality PC are developed in a coordinated fashion.

## Introduction

Kenyans with severe symptoms have limited access to palliative care (PC) trained healthcare workers (HCWs), especially in rural communities.^1,2,3,4^ In response, the Kenyan Ministry of Health has developed the Kenyan Palliative Care Policy 2021–2030 to serve as a roadmap for increasing access to PC.^2^ Included in this vision is delivery of PC services by all the members of the healthcare workforce,^5,6^ a goal that is in line with the World Health Organization (WHO) call to action for PC services within ‘primary healthcare and community/home-based care’.^7^

Developing primary PC as an integrated component of the healthcare system is the most expeditious path to improving access. In this model, all HCWs receive basic training to enable identification of patients with PC needs, then provide treatment or refer for specialist care.^7,8^ For current HCWs, virtual trainings such as Project ECHO^®^ have the advantage of improving HCW knowledge, job satisfaction and patient outcomes and decreasing both training costs and professional isolation.^9,10^

We initiated the Project ECHO^®^ Palliative Care for Western Kenya to foster primary PC in rural areas. The goal is to provide local HCWs with a forum for PC education and increased access to PC specialists. Project ECHO^®^ (Extension for Community Healthcare Outcomes) is a collaborative model of medical education and care management that was developed to improve clinical skills for HCWs practising outside of urban areas. It uses a virtual platform, and the sessions include case-based discussions, didactic lectures by subject matter experts and an opportunity for professionals to connect and collaborate.^11,12^

## Approach

**FIGURE 1:**
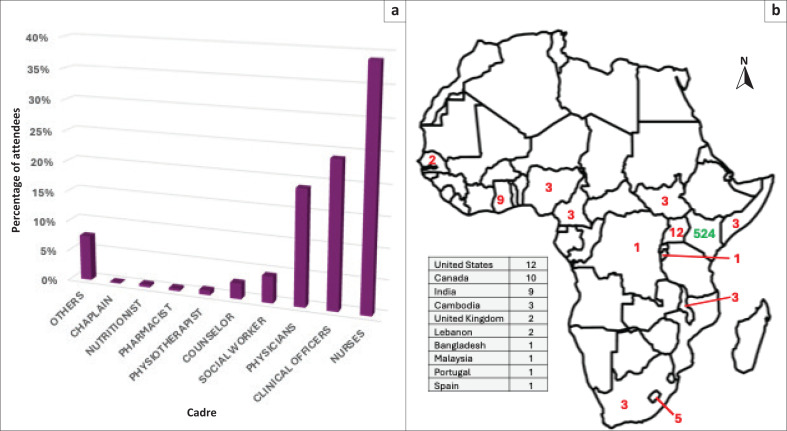
Participants’ information. (a) Attendees by cadre 2021–2023. (b) Location of attendees by country.

Our PC Project ECHO^®^, launched in 2021, offers 1-h monthly virtual sessions with didactic lectures and case discussions led by local and international experts. A nurse coordinator was appointed to manage the virtual platform, identification of primary care clinicians for case presentations, coordination with PC experts for didactic lecture presentations and collection of data. The Kenyan Hospice and Palliative Care Association (KEHPCA) supported with contacts for PC providers in Kenya. A WhatsApp group was created and used to advertise the sessions and link providers with experts. Emails were also used to send session invites. Primary care providers presented cases for discussion, after which PC experts presented didactic lectures. The majority of cases included discussion on symptom management in different types of advanced cancers, while a few cases included other medical conditions such as diabetes mellitus, end-stage renal disease, prematurity, hypoxic brain injury, hypoglycaemic brain injury, pulmonary tuberculosis and Hirschsprung disease. There were 12 didactic lectures each year following a curriculum on key PC topics (Table 1). The average time each participant spent online during a session was 51 minutes. Most participants were nurses and clinical officers (Figure 1a). Also in attendance were the allied health workers, which reflects the multidisciplinary approach to PC. The ECHO platform automatically surveys participants after each session and session materials are shared post-meeting.

**TABLE 1:** Didactic lecture topics.

Year 1	Year 2	Year 3
Principles of PC	Communication	PC needs in humanitarian crisis
The need of PC in Kenya	Breaking bad news	Assessing depression in advanced cancer
Pain management	Ethical principles	Tube feeding or not
Chronic non cancer pain management	Management of psychosocial burden	Terminal delirium
Breakthrough pain	Assessment and care of spiritual distress	Constipation in PC
Malignant wound management	Access to opioids at the county levels and narcotic use in Kenya	Diarrhoea in PC
Role of PC in COVID-19	Advance care planning	Determining prognosis in PC
Multiple myeloma: key aspects of diagnosis, management and supportive care	Human sexuality and PC	Hospice care
Paediatric oncology: survivorship and supportive care post treatment	Death and dying	Pressure ulcers I: staging and prevention
Management of nausea and vomiting during cancer treatment	Loss grief and bereavement	Pressure ulcers II: management
Anorexia and cachexia in chronic illness	Care of caregiver	Insomnia I: patient assessment
Refractory breathing problems in chronic lung disease	Urgent need of specialised independent PC departments in public health facilities	Insomnia II: management

COVID-19, coronavirus disease 2019; PC, palliative care.

## Relevant changes

Most participants (97%) strongly agreed or agreed that the sessions were useful. Similarly, participants felt the lectures and case discussions were helpful in improving their knowledge and skills. Importantly, participants strongly agreed or agreed that participation improved their professional confidence (95%) and reduced professional isolation (88%). Moreover, participants were extremely likely or likely to use the information learned in patient care (98%), and 96% would recommend the programme to a friend or colleague. Also, the discussion of the materials presented was rated very high or high by 93% of participants. There was steady growth in participation over the 3 years of the programme. Although the attendance was likely underreported because of group participation at clinical sites, the number of participants recorded in 2021, 2022 and 2023 was 446, 466 and 623, respectively.

In 2022, we began collecting geographic data and found an increased participation by individuals both within and outside Africa. In 2022, 94% of participants were from Kenya, and all but one of the other participants from Academic Model Providing Access to Healthcare (AMPATH; https://www.ampathkenya.org) members in North America. AMPATH Kenya is a collabortion between Moi University, Moi Teaching and Referral Hospital (MTRH) and a consortium of international medical schools. In 2023, the total number of participants increased, and attendees included a number of African and European countries (Figure 1b).

## Conclusion

### Lessons learnt

Virtual training is an attractive way to educate current HCWs on primary PC. Given the paucity of PC specialists in Kenya, curriculum development and implementation benefited from the engagement of KEHPCA and international collaborators. A dedicated coordinator was also important to maintain programme quality and connect with sites interested in participating. Using social networking has allowed us to reach would-be participants and allowed participants to promote the programme to others in their community. Having pre-formatted slides for the case presentation and reviewing the slides before the session has helped focus the discussion and keep the presentation within the allotted time. Sharing the case discussion and the didactic lecture with past participants fosters continuing education and ongoing engagement. The programme’s WhatsApp group also maintains engagement and is a likely factor in the reduced professional isolation reported by participants.

Our educational sessions highlighted clinical challenges for rural providers. A frequent issue raised by participants is lack of access to opioids. Through direct communication with PC specialists at AMPATH Kenya, or by connecting with others in our WhatsApp group, participants were able to identify the closest source for needed medications. Moreover, the ECHO programme established a connection with a PC specialist that has facilitated rapid access to emergent care, such as palliative radiation therapy for uncontrolled symptoms. We believe that the education programme led to the establishment of an ongoing connection between primary care providers and PC specialists and is the most important aspect of the training programme in terms of improved clinical care.

Kenya anticipates a marked increase in the need for PC services related to the growing population and an increasing incidence of chronic diseases. Public hospitals are also increasing access to advanced therapies such as chemotherapy, which can increase the complexity of symptom management. Our experience highlighted the need for primary PC to be developed in concert with expanding the number of PC specialists. For both HCWs in practice and those in training, there are inadequate PC specialists to meet the teaching need and an insufficient number of teaching hours allocated to PC training.^4,13^ Healthcare systems should consider concurrent and strategic investment in both primary and speciality PC to ensure primary PC providers have the support they need, and patients have access to speciality care when needed.
